# Supplementary material for: Detection of SARS-CoV-2 Derived Small RNAs and Changes in Circulating Small RNAs Associated with COVID-19
Source: Viruses. 2021 Aug 11;13(8):1593. doi: 10.3390/v13081593 (PMC8402885; doi:10.3390/v13081593)
Supplement: Supplementary file 1 [file viruses-13-01593-s001.zip › Suppl_Fig_SARS.pptx]

## Slide 1
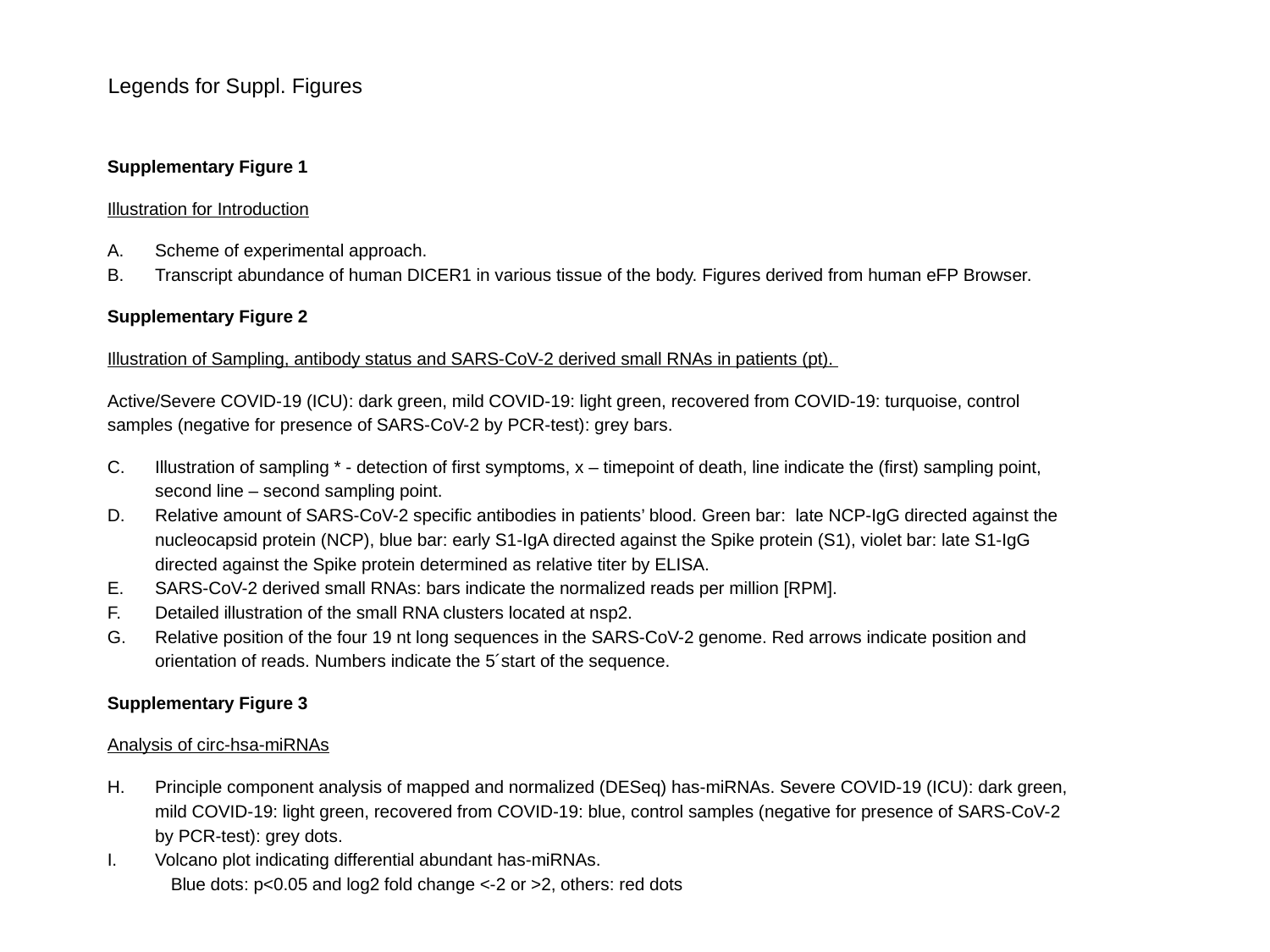

Legends for Suppl. Figures
Supplementary Figure 1
Illustration for Introduction
Scheme of experimental approach.
Transcript abundance of human DICER1 in various tissue of the body. Figures derived from human eFP Browser.
Supplementary Figure 2
Illustration of Sampling, antibody status and SARS-CoV-2 derived small RNAs in patients (pt).
Active/Severe COVID-19 (ICU): dark green, mild COVID-19: light green, recovered from COVID-19: turquoise, control samples (negative for presence of SARS-CoV-2 by PCR-test): grey bars.
Illustration of sampling * - detection of first symptoms, x – timepoint of death, line indicate the (first) sampling point, second line – second sampling point.
Relative amount of SARS-CoV-2 specific antibodies in patients’ blood. Green bar: late NCP-IgG directed against the nucleocapsid protein (NCP), blue bar: early S1-IgA directed against the Spike protein (S1), violet bar: late S1-IgG directed against the Spike protein determined as relative titer by ELISA.
SARS-CoV-2 derived small RNAs: bars indicate the normalized reads per million [RPM].
Detailed illustration of the small RNA clusters located at nsp2.
Relative position of the four 19 nt long sequences in the SARS-CoV-2 genome. Red arrows indicate position and orientation of reads. Numbers indicate the 5´start of the sequence.
Supplementary Figure 3
Analysis of circ-hsa-miRNAs
Principle component analysis of mapped and normalized (DESeq) has-miRNAs. Severe COVID-19 (ICU): dark green, mild COVID-19: light green, recovered from COVID-19: blue, control samples (negative for presence of SARS-CoV-2 by PCR-test): grey dots.
Volcano plot indicating differential abundant has-miRNAs.
Blue dots: p<0.05 and log2 fold change <-2 or >2, others: red dots

## Slide 2
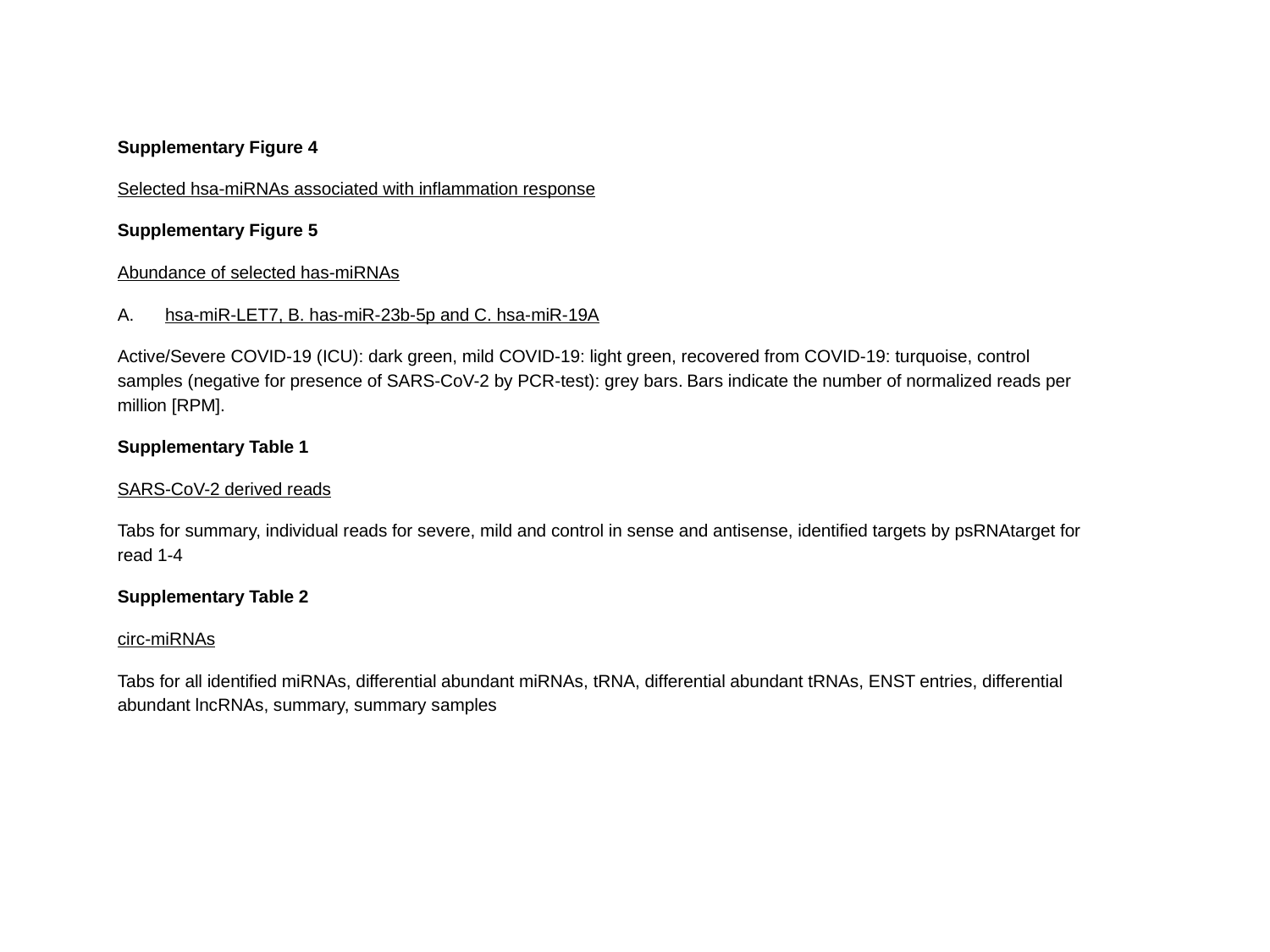

Supplementary Figure 4
Selected hsa-miRNAs associated with inflammation response
Supplementary Figure 5
Abundance of selected has-miRNAs
hsa-miR-LET7, B. has-miR-23b-5p and C. hsa-miR-19A
Active/Severe COVID-19 (ICU): dark green, mild COVID-19: light green, recovered from COVID-19: turquoise, control samples (negative for presence of SARS-CoV-2 by PCR-test): grey bars. Bars indicate the number of normalized reads per million [RPM].
Supplementary Table 1
SARS-CoV-2 derived reads
Tabs for summary, individual reads for severe, mild and control in sense and antisense, identified targets by psRNAtarget for read 1-4
Supplementary Table 2
circ-miRNAs
Tabs for all identified miRNAs, differential abundant miRNAs, tRNA, differential abundant tRNAs, ENST entries, differential abundant lncRNAs, summary, summary samples

## Slide 3
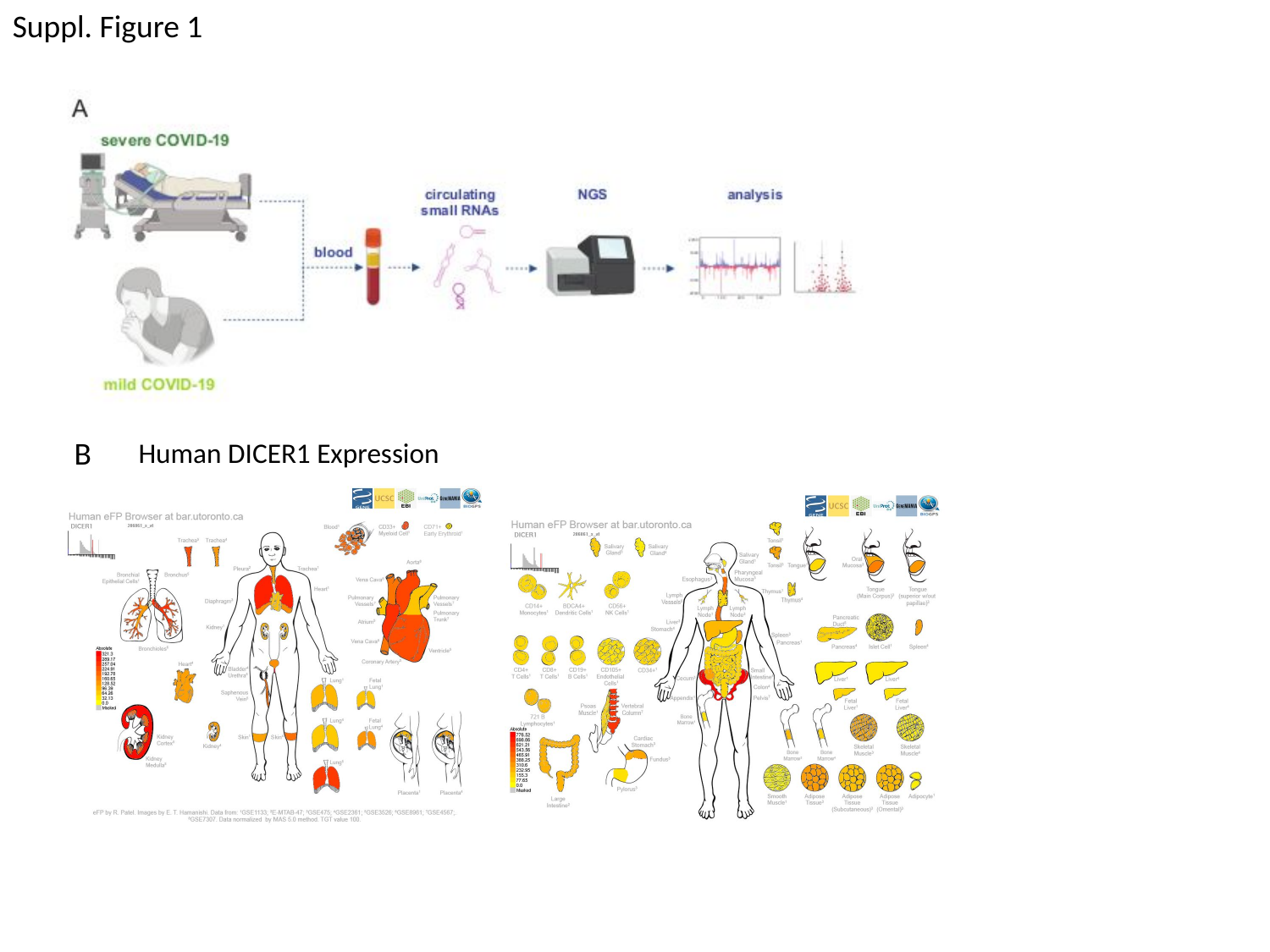

Suppl. Figure 1
B
Human DICER1 Expression

## Slide 4
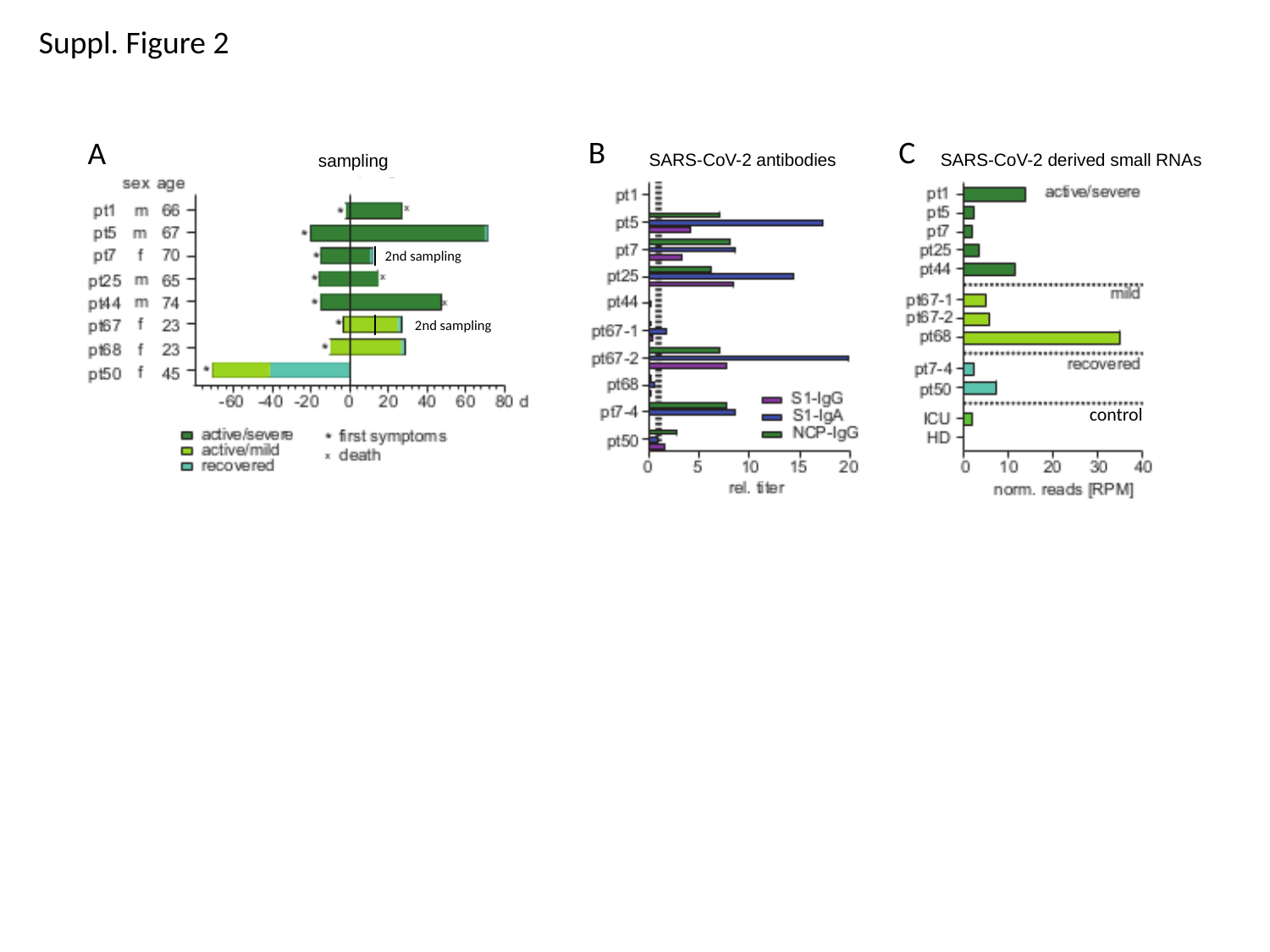

Suppl. Figure 2
2nd sampling
2nd sampling
control
B
C
A
SARS-CoV-2 antibodies
SARS-CoV-2 derived small RNAs
sampling

## Slide 5
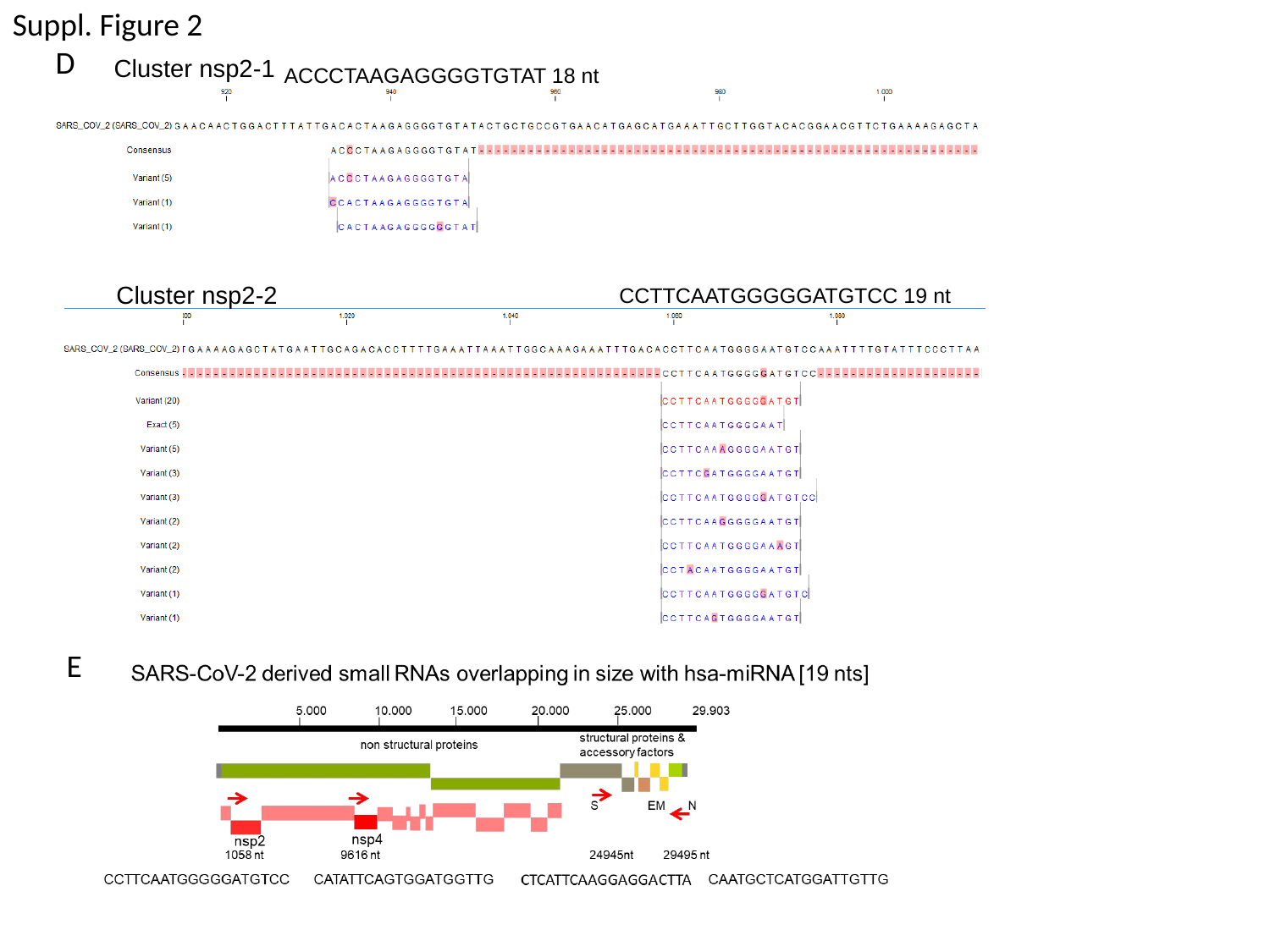

Suppl. Figure 2
D
Cluster nsp2-1
ACCCTAAGAGGGGTGTAT 18 nt
Cluster nsp2-2
CCTTCAATGGGGGATGTCC 19 nt
E

## Slide 6
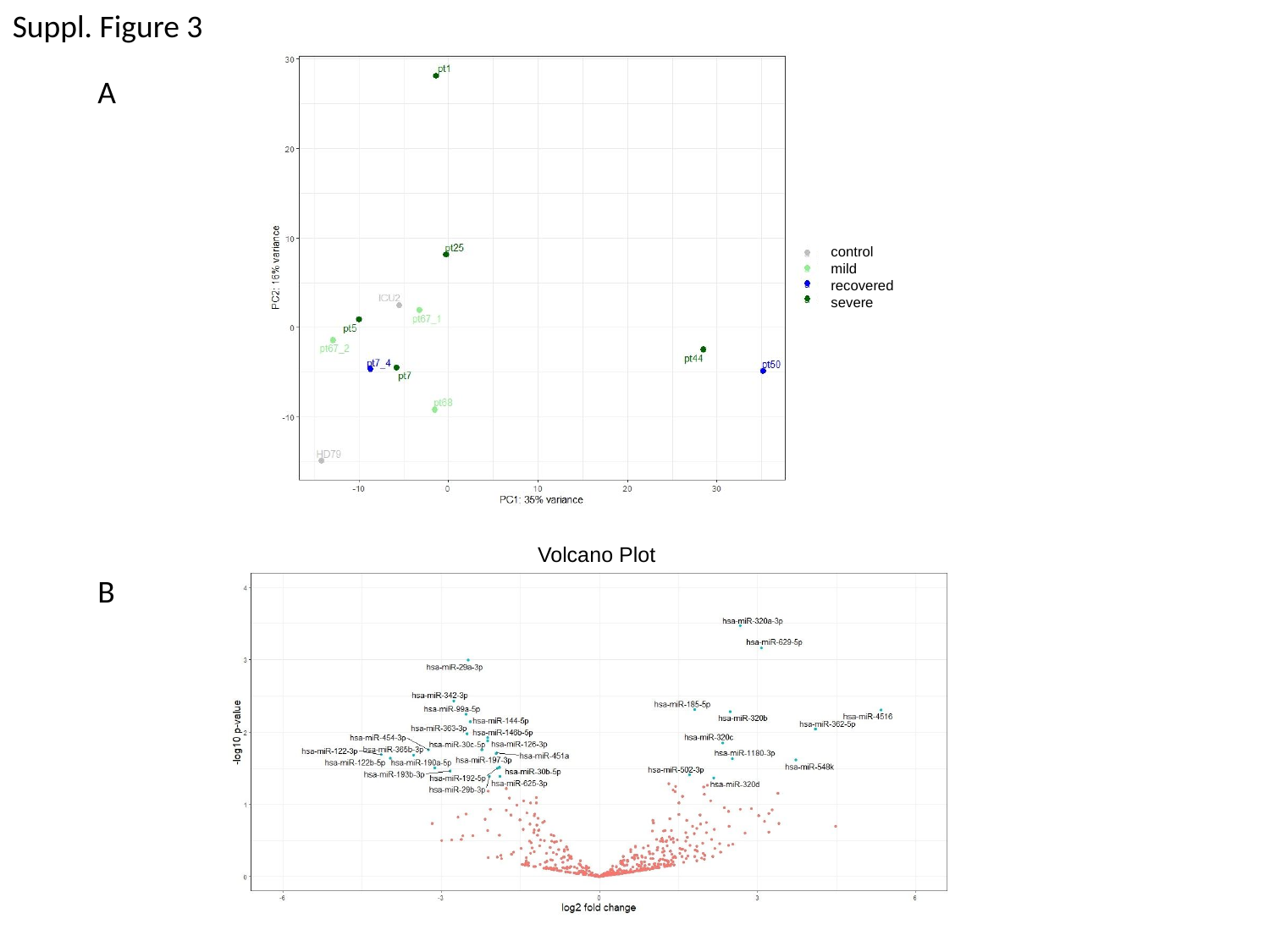

Suppl. Figure 3
control
mild
recovered
severe
A
Volcano Plot
B

## Slide 7
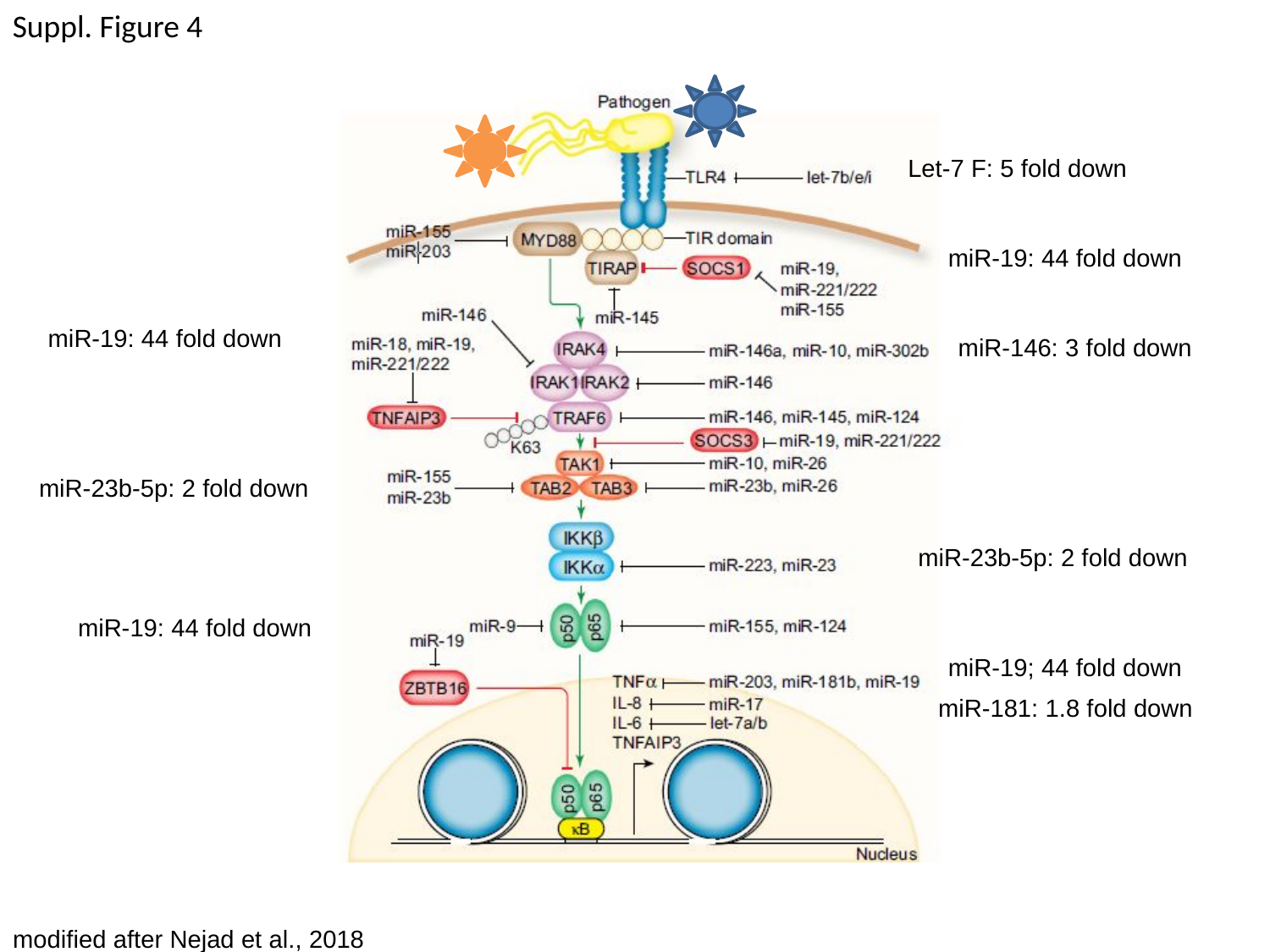

Suppl. Figure 4
Let-7 F: 5 fold down
miR-19: 44 fold down
miR-19: 44 fold down
miR-146: 3 fold down
miR-23b-5p: 2 fold down
miR-23b-5p: 2 fold down
miR-19: 44 fold down
miR-19; 44 fold down
miR-181: 1.8 fold down
modified after Nejad et al., 2018

## Slide 8
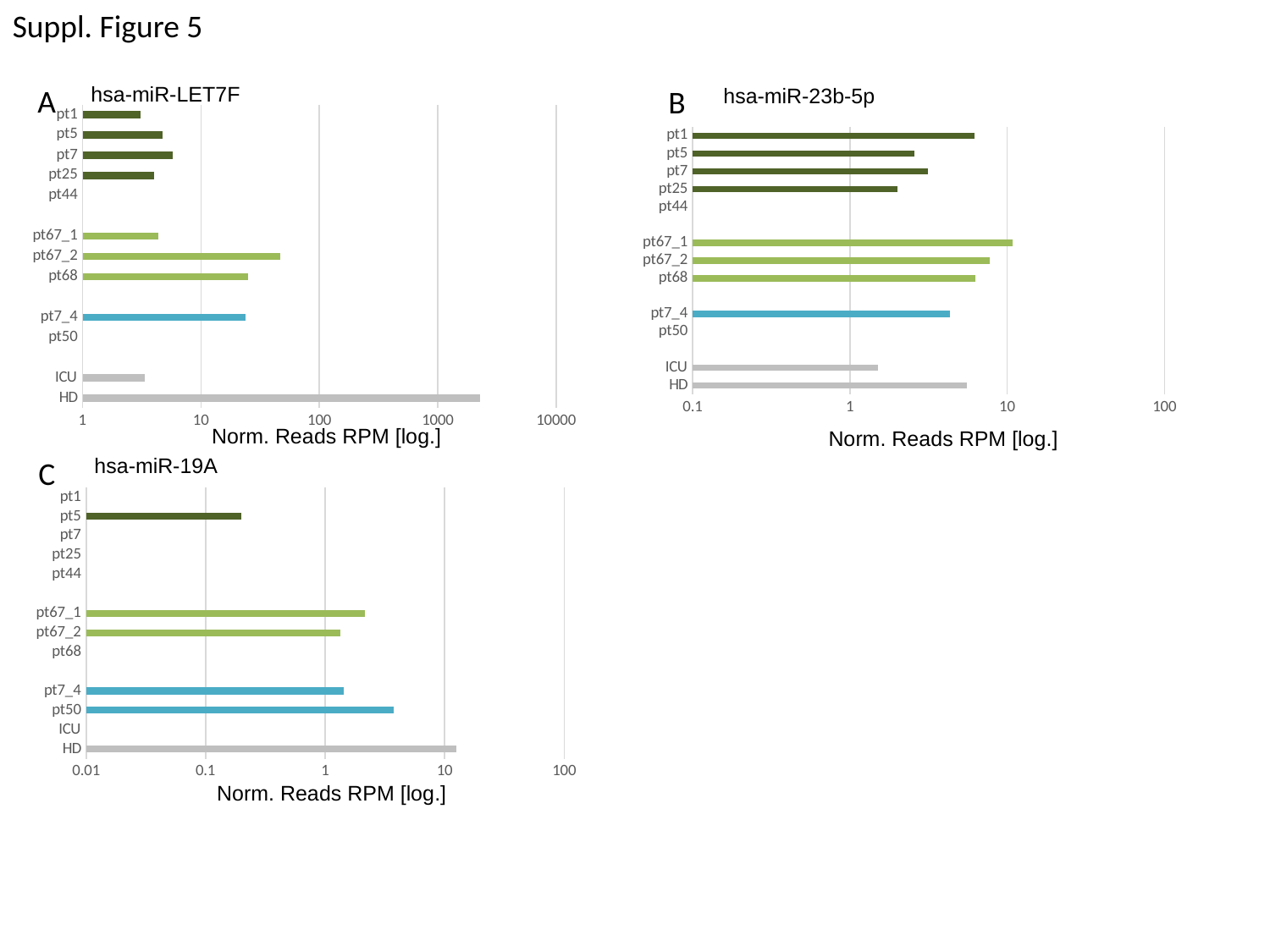

Suppl. Figure 5
hsa-miR-LET7F
A
B
hsa-miR-23b-5p
### Chart
| Category | |
|---|---|
| HD | 2297.08548664727 |
| ICU | 3.37629117662912 |
| | None |
| pt50 | 0.0 |
| pt7_4 | 23.9067196201655 |
| | None |
| pt68 | 25.166141868157 |
| pt67_2 | 46.8226000888889 |
| pt67_1 | 4.33606097202828 |
| | None |
| pt44 | 0.0 |
| pt25 | 4.00117607891357 |
| pt7 | 5.73174492245985 |
| pt5 | 4.74455396383024 |
| pt1 | 3.10109768793795 |
### Chart
| Category | |
|---|---|
| HD | 5.56531916813391 |
| ICU | 1.5005738562796 |
| | None |
| pt50 | 0.0 |
| pt7_4 | 4.30320953162979 |
| | None |
| pt68 | 6.29153546703926 |
| pt67_2 | 7.74762447513989 |
| pt67_1 | 10.8401524300706 |
| | None |
| pt44 | 0.0 |
| pt25 | 2.00058803945678 |
| pt7 | 3.12640632134173 |
| pt5 | 2.56996673040804 |
| pt1 | 6.2021953758759 |Norm. Reads RPM [log.]
Norm. Reads RPM [log.]
hsa-miR-19A
C
### Chart
| Category | hsa-miR-19A |
|---|---|
| HD | 12.5219681283013 |
| ICU | 0.0 |
| pt50 | 3.7531268415059 |
| pt7_4 | 1.43440317720993 |
| | None |
| pt68 | 0.0 |
| pt67_2 | 1.34741295219824 |
| pt67_1 | 2.16803048601414 |
| | None |
| pt44 | 0.0 |
| pt25 | 0.0 |
| pt7 | 0.0 |
| pt5 | 0.197689748492926 |
| pt1 | 0.0 |Norm. Reads RPM [log.]
